# Epigenetic and biological age acceleration in children with atopic dermatitis

**DOI:** 10.1016/j.jacig.2024.100275

**Published:** 2024-05-03

**Authors:** Richie Jeremian, Alexandra Malinowski, Edward S. Oh, Melinda Gooderham, Cathryn Sibbald, Jensen Yeung, Yuka Asai, Vincent Piguet, Carolyn S. Jack

**Affiliations:** a Faculty of Medicine & Health Sciences, McGill University, Montreal, Quebec, Canada; b McGill University Health Centre (MUHC) Center of Excellence for Atopic Dermatitis, Montreal, Quebec, Canada; c Campbell Family Mental Health Research Institute, Centre for Addiction and Mental Health, Toronto, Ontario, Canada; d SKiN Centre for Dermatology, Peterborough, Probity Medical Research, Waterloo; and Queen’s University, Kingston, Ontario, Canada; e Department of Paediatrics, Division of Dermatology, Temerty Faculty of Medicine, University of Toronto, Toronto, Ontario, Canada; f Department of Medicine, Division of Dermatology, Temerty Faculty of Medicine, University of Toronto, Toronto, Ontario, Canada; g Division of Dermatology, School of Medicine, Faculty of Health Sciences, Queen’s University, Kingston, Ontario, Canada; h Department of Medicine, Division of Dermatology, Women’s College Hospital, Toronto, Ontario, Canada

**Keywords:** Atopic dermatitis, DNA methylation, epigenetic aging, biological aging, inflammatory biomarkers

## Abstract

**Background:**

Atopic dermatitis (AD) is a chronic inflammatory skin disease resulting from the complex interplay of genetic and environmental factors, meriting exploration using temporally dynamic biomarkers. DNA methylation–based algorithms have been trained to accurately estimate biological age, and deviation of predicted age from true age (epigenetic age acceleration) has been implicated in several inflammatory diseases, including asthma.

**Objective:**

We sought to determine the role of epigenetic and biological aging, telomere length, and epigenetically inferred abundance of 7 inflammatory biomarkers in AD.

**Methods:**

We performed DNA methylation–based analyses in a pediatric AD cohort (n = 24, mean ± standard deviation [SD] age 2.56 ± 0.28 years) and age-matched healthy subjects (n = 24, age 2.09 [0.15] years) derived from blood using 5 validated algorithms that assess epigenetic age (Horvath, Skin&Blood) and biological age (PhenoAge, GrimAge), telomere length (TelomereLength), and inflammatory biomarker levels.

**Results:**

Epigenetic and biological age, but not telomere length, were accelerated in AD patients for 4 algorithms: Horvath (+0.88 years; 95% confidence interval [CI], 0.33 to 1.4; *P* = 2.3 × 10^−3^), Skin&Blood (+0.95 years; 95% CI, 0.67 to 1.2; *P* = 1.8 × 10^−8^), PhenoAge (+8.2 years; 95% CI, 3.4 to 13.0; *P* = 1.3 × 10^−3^), and GrimAge (+1.8 years 95% CI, 0.22 to 3.3; *P* = .026). Moreover, patients had increased levels of β_2_ microglobulin (+47,584.4 ng/mL; *P* = .029), plasminogen activation inhibitor 1 (+3,432.9 ng/mL; *P* = 1.1 × 10^−5^), and cystatin C (+31,691 ng/mL; *P* = 4.0 × 10^−5^), while levels of tissue inhibitor metalloproteinase 1 (−370.7 ng/mL; *P* = 7.5 × 10^−4^) were decreased compared to healthy subjects.

**Conclusion:**

DNA methylation changes associated with epigenetic and biological aging, and inflammatory proteins appear early in life in pediatric AD and may be relevant clinical biomarkers of pathophysiology.

Atopic dermatitis (AD), a chronic inflammatory skin disorder that is highly heterogeneous in its clinical presentation, is a product of environmental and genetic risk factors manifesting in a temporally variable manner.^1^ Large gaps remain in our understanding of this relationship, and genome-wide association studies are unable to account for pathologic dysregulation of gene expression in AD. Epigenetic modifications act at the interface of the environment and genome, change in response to exposures across the life-span, and have been shown to be reflective of immune system dysregulation, autoimmunity, and promotion of a proinflammatory milieu in the context of AD and other inflammatory skin diseases.^2^ Thus, improving understanding of the disease requires comprehensive investigations with tools that assess the influence of dynamic changes to gene function.

Recently, so-called epigenetic clock algorithms have garnered significant attention in chronic disease research as a result of their ability to accurately predict age and telomere length. These algorithms are trained on large-scale data sets of human tissues and have identified that DNA methylation (DNAm) of specific cytosine–guanine dinucleotide (CpG) sites is associated with age and disease.^3^ Although trained using distinct data sets, these algorithms are collectively enriched for CpGs found within pathways involved in tissue development and differentiation, cell proliferation, and proinflammatory signaling pathways (including cytokine signaling pathways, response to IFN-γ, TNF-mediated signaling, and the Janus activating kinase–signal transducer and activator of transcription [aka JAK-STAT] cascade).^4^, ^5^, ^6^, ^7^ Deviation of epigenetic (molecular) from chronological (true numerical) age, termed epigenetic age dysregulation (acceleration and deceleration), has been observed in numerous complex diseases and syndromes,^3^^,^^4^ including inflammatory diseases such as chronic kidney disease,^8^ inflammatory bowel disease,^9^ primary sclerosing cholangitis,^10^ and hidradenitis suppurativa.^11^

DNAm-based aging has not been studied in the context of AD. To this end, we utilized a public data set of peripheral blood DNAm from pediatric participants, leveraging 5 validated clock algorithms to investigate the role of epigenetic, biological, and telomere-linked age acceleration in AD patients compared to healthy subjects. The selected clocks are among the most widely used and have been trained on large data sets and rigorously validated through independent testing.^5^^,^^12^ These include first-generation clocks, including Horvath, a pan-tissue estimator developed using 8,000 samples encompassing 51 healthy tissues and cell types,^2^ and its updated counterpart, Skin&Blood, trained using skin and blood tissue that outperforms Horvath in its accuracy to predict chronological age based on DNAm state (with the outputs of both clocks known as epigenetic age).^6^ Second-generation clocks (PhenoAge and GrimAge) are trained to predict aging-related physiologic processes such as inflammation, biomarker status, and immune function, collectively measuring physiologic function and risk of all-cause mortality (referred to as biological age).^3^ The latter, GrimAge, is a composite of DNA-based surrogates of 7 plasma proteins and smoking pack years.^7^ We also included a DNAm-based estimator of telomere length (TelomereLength), which decreases with age through a phenomenon associated with aging-related diseases.^13^

## Methods

Study design and reporting followed “STrengthening the REporting of Genetic Association Studies (STREGA): An Extension of the STROBE Statement.”^14^

### Participant cohort and DNAm data

Preprocessed peripheral blood DNAm data from a pediatric patient cohort of moderate-to-severe AD patients (n = 24; 17 male, 5 female; mean ± standard deviation [SD]) age 2.56 ± 0.28 years) and healthy subjects (n = 24; 12 male, 12 female; age 2.09 ± 0.15 years), based on the Illumina HumanMethylation450 microarray, were obtained from the Gene Expression Omnibus (www.ncbi.nlm.nih.gov/geo, GSE152084). Informed consent was obtained from participants’ parents/guardians. AD diagnosis was based on Hanifin and Rajka criteria, where family history of atopic disease is a major criterium, and disease severity was assessed using the Severity Scoring of Atopic Dermatitis index. Blood and IgE samples were taken before treatment. Healthy subjects were included from a group of pediatric patients with no personal or family history of AD. AD patients had a mean ± SD Severity Scoring of Atopic Dermatitis score of 31.24 ± 3.67, indicating moderate disease, and serum IgE level 1,136.3 ± 218.3 IU/mL. Additional cohort characterization and data processing are described in full by Chen et al.^15^

### Estimation of epigenetic and biological age, telomere length, and plasma protein levels

Epigenetic age was investigated using the validated University of California, Los Angeles–based DNA Methylation Age Calculator (dnamage.genetics.ucla.edu/new) for 5 clock algorithms (Horvath, Skin&Blood, PhenoAge, GrimAge, and DNAmTL, denoted here as TelomereLength) that compute age-adjusted measures of epigenetic age using Elastic Net regression of methylation values of specific CpGs on chronological age.^16^ Two of these clocks, Horvath (353 CpGs, trained using DNA from various human tissues of healthy subjects and multiple disease groups)^3^ and Skin&Blood (391 CpGs, trained on skin and blood DNA),^17^ were designed to predict chronological age. In contrast, the PhenoAge and GrimAge clocks (513 and 1,030 CpGs, respectively, trained on whole blood DNA) incorporate several clinical and aging biomarkers; they were designed to predict morbidity, all-cause mortality, and health span, collectively termed biological age.^6^^,^^7^ In PhenoAge, these biomarkers include albumin, creatinine, serum glucose, C-reactive protein, alkaline phosphatase, lymphocyte percentage, mean cell volume, red cell distribution width, and white blood cell count. GrimAge incorporates measures of 7 plasma proteins (adrenomedullin, β_2_ microglobulin, cystatin C, growth differentiation factor 15, leptin, plasminogen activation inhibitor 1 [PAI-1], tissue inhibitor metalloproteinase 1 [TIMP-1]); estimated serum levels (ng/mL, inferred from DNAm state) of these proteins were analyzed. TelomereLength (140 CpGs, trained using data sets of whole and peripheral blood) is trained to predict biological age by estimating telomere length (kb), which is known to decrease across the life-span.^13^

### Statistical analysis

Association between chronological and epigenetic age was computed by Pearson product–moment correlation *(r)*. Epigenetic age acceleration was computed as the case–control difference in mean residual values derived from the clock-specific regression of DNAm on chronological age. Age acceleration as well as case–control differences in GrimAge-related plasma protein estimates were assessed by Welch *t* test. Serum IgE levels collected from the disease group were compared to all generated variables by Pearson product–moment correlation *(r)*. Analyses were further stratified by sex; and by IgE-low (n = 12; 9 male, 3 female) and IgE-high (n = 12; 10 male, 2 female) using 500 IU/mL as the threshold. Correlations underwent correction for multiple testing using the false discovery rate method.^8^

## Results

In this study, we utilized a public DNAm data set from Chen et al^15^ to test for differences in epigenetic age between pediatric AD patients and matched healthy subjects using 5 clock algorithms. We did not detect a significant difference in chronological age between AD patients and healthy subjects (*P* > .05), thus validating appropriate age matching in this cohort (Table I).

**Table I:** Overview of patient cohort

Characteristic	AD	AD with:		Healthy subjects
		IgE-high	IgE-low	
No. of subjects (no. female)	24 (5)	12 (2)	12 (3)	24 (12)
Age (years), mean ± SD	2.6 ± 1.4	3.1 ± 1.1	2.1 ± 1.5	2.1 ± 0.8
Sex				
Male	2.5 (1.5)	2.9 (1.1)	2.0 (1.7)	1.9 (0.7)
Female	3.0 (1.0)	4.1 (0.4)	2.3 (0.3)	2.3 (0.8)
Serum IgE (IU/mL), mean ± SD	736.8 ± 898.6	1,437.2 ± 785.7	36.5 ± 30.2	24.6 ± 6.9
Male	692.8 ± 801.5	1,280.6 ± 688.0	39.6 ± 31.4	NA
Female	904.3 ± 1,306.9	2,220 ± 1,029.6	27.2 ± 29.9	NA

*NA*, Not applicable.

We investigated differences in epigenetic and biological age, as well as telomere length, between pediatric AD patients and matched healthy subjects using 5 DNAm-based clock algorithms. We first investigated the accuracy of the epigenetic clocks in predicting chronological age; we observed moderate-to-strong, significant correlation between chronological and DNAm age for all tested clocks in both disease and healthy subject groups (Table II; Horvath, AD *r* = 0.84, *P* = 3.8 × 10^−7^, healthy *r* = 0.69, *P* = 5.1 × 10^−4^; Skin&Blood, AD *r* = 0.96, *P* = 1.8 × 10^−13^, healthy *r* = 0.76, *P* = 7.4 × 10^−5^; PhenoAge, AD *r* = 0.86, *P* = 1.7 × 10^−7^, healthy *r* = 0.49, *P* = .022; GrimAge AD, *r* = 0.70, *P* = 1.5 × 10^−4^, healthy *r* = 0.44, *P* = .032; TelomereLength, AD *r* = −0.77, *P* = 1.3 × 10^−5^, healthy *r* = −0.48, *P* = .023). We observed comparable findings among male participants in both AD (−0.83 > *r* > 0.80, *P* = 2.0 × 10^−12^ ∼ 4.5 × 10^−5^) and healthy subjects (−0.58 > *r* > 0.61, *P* = 4.6 × 10^−5^ ∼ .049). Among female subjects, we only found a significant correlation in healthy subjects for the Horvath clock (*r* = 0.68, *P* = .034). When considering IgE levels, we observed strong, significant correlation between chronological and DNAm age for all clocks among all IgE-low patients (−0.89 > *r* > 0.78, *P* = 6.1 × 10^−8^ ∼ 2.5 × 10^−3^) and male subjects in this group (−0.92 > *r* > 0.80, *P* = 2.1 × 10^−7^ ∼ .010). In patients with elevated IgE, significant age correlation was only noted for the Skin&Blood and PhenoAge clocks among all (*r* = 0.72-0.92, *P* = 1.2 × 10^−4^ to .021) and male-only (*r* = 0.70-0.94, *P* = 2.4 × 10^−4^ to .039) participant groups. As expected, the direction of correlation was positive for all tested clocks except TelomereLength, which is known to decrease across the life-span. These findings demonstrate the accuracy of the chosen clocks in predicting chronological age in this cohort, enabling downstream case–control comparisons.

**Table II:** Pearson correlations *(r)* of chronological age, epigenetic age, and telomere length using 5 DNAm-based clock algorithms stratified by disease status, sex, and serum IgE level

Clock algorithm	AD			Healthy subjects		
	Pearson *r*	*q* value	95% CI	Pearson *r*	*q* value	95% CI
All participants						
Horvath	0.843	3.80E-07	0.67 to 0.93	0.69	5.15E-04	0.39 to 0.85
Skin&Blood	0.964	1.77E-13	0.92 to 0.98	0.76	7.40E-05	0.52 to 0.89
PhenoAge	0.861	1.66E-07	0.70 to 0.94	0.49	2.26E-02	0.10 to 0.74
GrimAge	0.697	1.52E-04	0.41 to 0.86	0.44	3.16E-02	0.044 to 0.72
TelomereLength	−0.770	1.34E-05	−0.90 to −0.53	−0.48	2.26E-02	−0.74 to −0.09
All participants (male)						
Horvath	0.87	1.78E-06	0.70 to 0.95	0.77	8.73E-03	0.35 to 0.93
Skin&Blood	0.98	1.96E-12	0.94 to 0.99	0.93	4.61E-05	0.77 to 0.98
PhenoAge	0.88	1.51E-06	0.71 to 0.95	0.70	1.77E-02	0.22 to 0.91
GrimAge	0.80	4.53E-05	0.54 to 0.92	0.61	4.48E-02	0.053 to 0.88
TelomereLength	−0.83	1.46E-05	−0.93 to −0.60	−0.58	4.92E-02	−0.86 to −0.006
All participants (female)						
Horvath	NS			0.68	3.49E-02	0.18 to 0.90
Skin&Blood	NS				NS	
PhenoAge	NS				NS	
GrimAge	NS				NS	
TelomereLength	NS				NS	
IgE-high (all)				—		
Horvath		NS				
Skin&Blood	0.92	1.23E-04	0.73 to 0.98			
PhenoAge	0.72	2.08E-02	0.25 to 0.92			
GrimAge		NS				
TelomereLength		NS				
IgE-high (male)				—		
Horvath		NS				
Skin&Blood	0.94	2.37E-04	0.77 to 0.99			
PhenoAge	0.70	3.86E-02	0.13 to 0.92			
GrimAge		NS				
TelomereLength		NS				
IgE-high (female)∗	—			—		
Horvath						
Skin&Blood						
PhenoAge						
GrimAge						
TelomereLength						
IgE-low (all)				—		
Horvath	0.95	4.03E-06	0.84 to 0.99			
Skin&Blood	0.98	6.10E-08	0.94 to 0.99			
PhenoAge	0.90	1.16E-04	0.67 to 0.97			
GrimAge	0.78	2.51E-03	0.38 to 0.94			
TelomereLength	−0.89	1.16E-04	−0.97 to −0.65			
IgE-low (male)				—		
Horvath	0.97	2.93E-05	0.87 to 0.99			
Skin&Blood	0.99	2.12E-07	0.97 to 0.99			
PhenoAge	0.95	1.12E-04	0.79 to 0.99			
GrimAge	0.80	9.67E-03	0.29 to 0.96			
TelomereLength	−0.92	4.58E-04	−0.98 to −0.67			
IgE-low (female)						
Horvath	NS			—		
Skin&Blood						
PhenoAge						
GrimAge						
TelomereLength						

Analyses are adjusted for multiple testing correction by false discovery rate method. *Dash* indicates not applicable; *NS,* not significant.

∗ Insufficient sample size for analyses.

**Fig 1:**
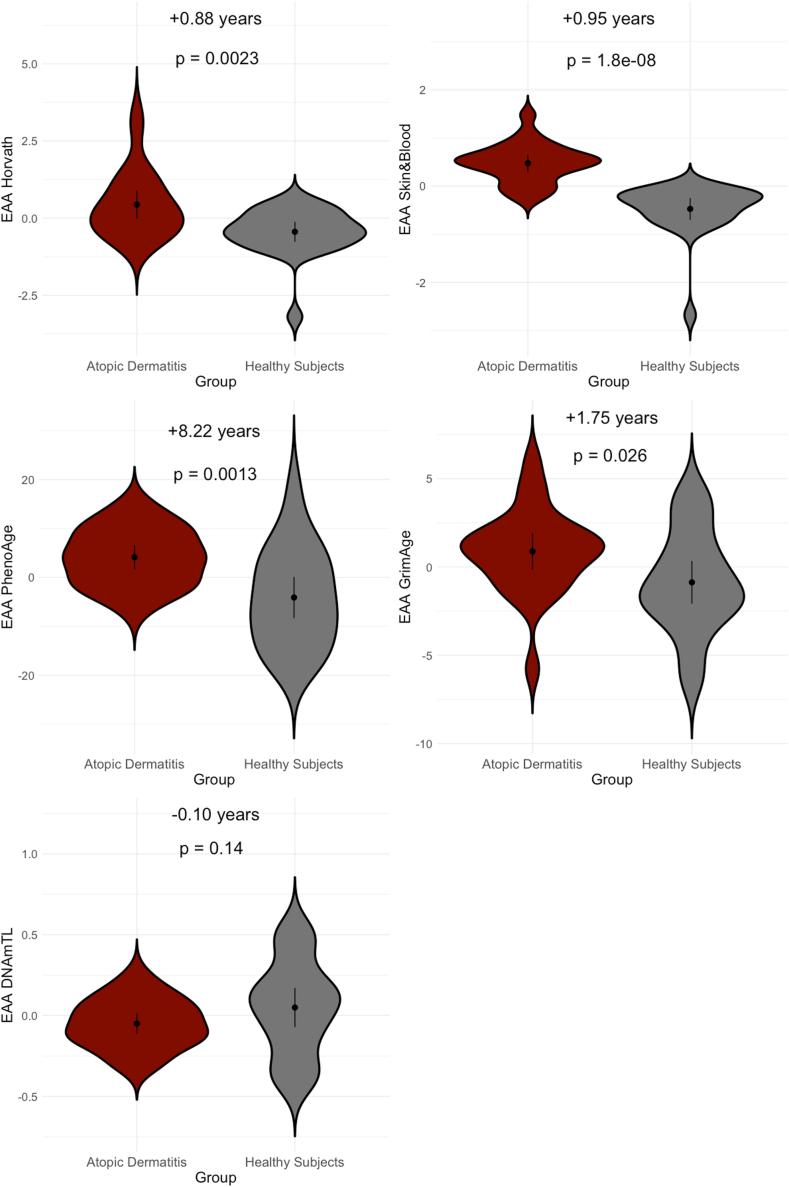
Epigenetic age acceleration (EAA) in AD compared to healthy subjects across 5 epigenetic clock algorithms. Y-axes represent age-adjusted measures of epigenetic age acceleration (years) and telomere length (kb). *P* values reflect 2-tailed groupwise differences in mean acceleration measures between groups by Welch *t* test; *point* and *whiskers* represent mean group value and 95% CI, respectively.

We then tested for case–control differences in epigenetic and biological age acceleration (Fig 1) and observed significant acceleration in AD patients versus healthy subjects for 4 clocks: Horvath (+0.88 years; 95% confidence interval [CI], 0.33 to 1.4; *P* = 2.3 × 10^−3^), Skin&Blood (+0.95 years; 95% CI, 0.67 to 1.2; *P* = 1.8 × 10^−8^), PhenoAge (+8.2 years; 95% CI, 3.4 to 13.0; *P* = 1.3 × 10^−3^), and GrimAge (+1.8 years 95% CI, 0.22 to 3.3; *P* = .026). In contrast, we did not observe a significant difference in TelomereLength between AD and healthy control (*P* > .05). We also investigated sex differences in DNAm-based aging, observing significantly greater epigenetic age acceleration in male versus female subjects in the IgE-high group for the Horvath (+1.6 years; 95% CI, 0.35 to 2.8; *P* = .02) and Skin&Blood (+0.71 years, 95% CI, 0.27 to 1.1, *P* = 7.8 × 10^−3^) clocks. Interestingly, we found significantly lower TelomereLength (−0.15 kilobases; 95% CI, −0.058 to −0.24; *P* = 4.7 × 10^−3^) among male, versus female, IgE-low patients. No other sex-based differences in either patients or healthy subjects were observed. Using a multiclock strategy, we demonstrated that epigenetic (Horvath, Skin&Blood) and biological (PhenoAge, GrimAge) age are accelerated in AD patients compared to healthy subjects, with no changes to telomere length.

**Fig 2:**
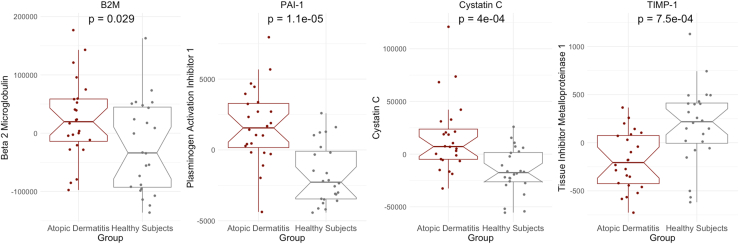
Cross-sectional differences in GrimAge-associated estimated plasma proteins in AD patients. Y-axes represent age-adjusted relative estimated abundance of plasma protein levels (ng/mL) inferred from DNAm state. *P* values reflect 2-tailed groupwise differences by Welch *t* test. *Notches* represent group median values; *boxes,* interquartile range; and *whiskers,* range of extreme values.

We next compared groupwise estimates of GrimAge-associated plasma proteins and observed significant differences in estimated levels of 4 of 7 GrimAge proteins in AD patients versus healthy subjects. As shown in Fig 2, increased estimated levels of β_2_ microglobulin (+47,584.4 ng/mL; *P* = .029), PAI-1 (+3,432.9 ng/mL; *P* = 1.1 × 10^−5^), and cystatin C (+31,691 ng/mL; *P* = 4.0 × 10^−5^) were found in AD patients, while TIMP-1 levels (−370.7 ng/mL; *P* = 7.5 × 10^−4^) were decreased. Surprisingly, among AD patients, PAI-1 levels were significantly lower in the IgE-high versus IgE-low patient group (−2,797.6 ng/mL; *P* = 6.2 × 10^−3^). After adjusting for multiple comparisons, we did not observe significant associations between serum IgE level, epigenetic age acceleration, or GrimAge-based protein composition, suggesting that each outcome is indecently associated with AD.

## Discussion

This study demonstrates that significant alterations in DNAm, which is associated with aging and other type 2 inflammatory diseases, are found as early as 2 years of age in AD patients. Using 5 independently designed clock algorithms, epigenetic and biological age acceleration was observed in DNAm of blood from pediatric AD patients, an effect that was not attributable to changes in telomere length. This distinction is concordant with *in vitro* data demonstrating a distinction between epigenetic and biologic age acceleration, and telomere attrition.^5^ Our findings are supported by 2 recent studies that observed epigenetic age acceleration using the Horvath clock in 2 distinct childhood cohorts of asthma and allergy, associated with greater odds of atopic sensitization, environmental and food allergen sensitization, greater total serum IgE levels, and response to systemic corticosteroids.^18^^,^^19^ These findings—in asthma, allergy, and now AD—support a role for environmental drivers and altered DNAm early in atopic disease. We also observed accelerated epigenetic and biological age and shortened telomere length in male versus female AD patients. However, this finding was only observed among 2 subsets of participants and may reflect known sex differences in DNAm-based aging, with male subjects showing acceleration in all age groups.^4^

Using the GrimAge clock, which utilizes DNAm-based surrogates of aging-related biomarkers, we identified significant differences in 4 of 7 plasma proteins in AD patients. These include elevated PAI-1 levels, polymorphisms of which have been found to associated with increased risk of asthma and IgE-mediated allergic rhinitis.^20^^,^^21^ This serine protease inhibitor is an essential component of the fibrinolytic system, but it also plays a role in cell migration and inflammation, notably in airway remodeling and hyperresponsiveness.^22^^,^^23^ PAI-1 may contribute to T_H_2 cell responses, given its upregulation by IL-4 and IL-13 in macrophages,^24^ along with its capacity to inhibit T_H_1 responses^25^ and enhance IL-4–induced thymic stromal lymphopoietin in airway epithelial cells.^26^ Dupilumab, a biologic treatment for both AD and asthma, reduced PAI-1 expression in AD skin.^27^ We postulate that PAI-1 may be involved at an early phase of disease, and its mechanistic link to IgE expression requires further investigation. The GrimAge clock also predicted higher expression of cystatin C in AD. Cystatin C is a biomarker of renal function^28^ but is also associated with inflammation^29^ and asthma.^30^ Notably, cystatin C is strongly upregulated in the granular layer of acral keratinocytes and associated with the downregulation of filaggrin and keratin citrullination enzymes.^31^ The JAK1/ 2 inhibitor baricitinib was found to reduce cystatin C levels in AD patients after 4 weeks.^32^ In contrast, GrimAge predicted reduced levels of TIMP-1 in the AD cohort. AD skin lesions are known to have lower levels of TIMP-1 and TIMP-2 along with increased matrix metalloproteinase activity,^33^ and TIMP-1 is downregulated in IL-4– and IL-13–polarized macrophages.^24^ TIMP-1 regulates matrix metalloproteinases and their inhibitors, and its elevation is associated with lichenification and prurigo in AD.^34^ Collectively, these findings highlight a novel method to test for biomarkers in AD patients. It merits further exploration to validate DNAm findings directly at the messenger RNA and protein levels.

Given the nature of public data, we were not able to obtain more extensive clinical information and disease measures. While our findings may be influenced by unknown coexisting atopic comorbidities, the risk is minimized by young patient age: asthma and allergic rhinitis develop later in life in most cases.^35^ We were constrained by a small sample size biased by male participants and no ethnic diversity. Given the exploratory nature of the study, the low participant diversity may have added power for detecting significant case–control differences. Epigenetic age prediction was less reliable among female participants and in the IgE-high group, which could be explained by relatively smaller sample size of these individuals, or perhaps may be a reflection of *bona fide* sex differences in epigenetic age. Despite these trends, the implemented clocks have been shown to be reliable in both sexes. To overcome these limitations, studies that encompass much larger and more diverse patient cohorts, more detailed clinical data capturing outcomes, skin as well as blood samples, validating methodologies (flow cytometry, RNA sequencing, proteomics), and longitudinal designs are needed.

In summary, DNAm and epigenetic aging analyses hold promise as complementary tools to study proinflammatory immune dysregulation in AD. This study provides early findings supporting the hypothesis of accelerated epigenetic aging in a cohort of childhood-onset AD, which have the potential to elucidate molecular and pathophysiologic insights that will improve understanding of the AD disease process across the life-span. Future studies correlating epigenetic clock metrics with clinical data will demonstrate the true potential of this biomarker and provide nuanced insights into the synergistic effect of causal factors on the “missing heritability” of AD.

**Key messages**

• We report epigenetic and biological age acceleration across 4 DNAm-based clocks in a pediatric AD cohort.
• We observe differential levels of 4 inflammatory proteins previously shown to be implicated in type 2 immune responses.
• We demonstrate for the first time that epigenetic changes associated with biological aging and differential expression of novel biomarkers appear early in life among AD patients.

## Disclosure statement

This work was supported by funding from the 10.13039/100008582McGill University Faculty of Medicine & Health Sciences, the McGill University Health Centre Foundation, and the International Society of Atopic Dermatitis.

Disclosure of potential conflict of interest: The authors declare that they have no relevant conflicts of interest.
